# Invasive Methicillin-Resistant Staphylococcus aureus USA500 Strains from the U.S. Emerging Infections Program Constitute Three Geographically Distinct Lineages

**DOI:** 10.1128/mSphere.00571-17

**Published:** 2018-05-02

**Authors:** M. B. Frisch, S. Castillo-Ramírez, R. A. Petit, M. M. Farley, S. M. Ray, V. S. Albrecht, B. M. Limbago, J. Hernandez, I. See, S. W. Satola, T. D. Read

**Affiliations:** a Division of Infectious Diseases, Department of Medicine, Emory University School of Medicine, Atlanta, Georgia, USA; b Center for Genomic Sciences, UNAM, Cuernavaca, Mexico; c Division of Healthcare Quality Promotion, Centers for Disease Control and Prevention, Atlanta, Georgia, USA; d Atlanta VA Medical Center, Decatur, Georgia, USA; University of Nebraska Medical Center

**Keywords:** evolution, IS*256*, MRSA, USA300, adenosine, drug resistance

## Abstract

USA500 isolates are clonal complex 8 (CC8) Staphylococcus aureus strains closely related to the prominent community- and hospital-associated USA300 group. Despite being relatively understudied, USA500 strains cause a significant burden of disease and are the third most common methicillin-resistant S. aureus (MRSA) strains identified in the U.S. Emerging Infections Program (EIP) invasive S. aureus surveillance. To better understand the genetic relationships of the strains, we sequenced the genomes of 539 USA500 MRSA isolates from sterile site infections collected through the EIP between 2005 and 2013 in the United States. USA500 isolates fell into three major clades principally separated by their distribution across different U.S. regions. Clade C1 strains, found principally in the Northeast, were associated with multiple IS*256* insertion elements in their genomes and higher levels of antibiotic resistance. C2 was associated with Southern states, and E1 was associated with Western states. C1 and C2 strains all shared a frameshift in the gene encoding AdsA surface-attached surface protein. We propose that the term “USA500” should be used for CC8 strains sharing a recent common ancestor with the C1, C2, and E1 strains but not in the USA300 group.

**IMPORTANCE** In this work, we have removed some of the confusion surrounding the use of the name “USA500,” placed USA500 strains in the context of the CC8 group, and developed a strategy for assignment to subclades based on genome sequence. Our new phylogeny of USA300/USA500 will be a reference point for understanding the genetic adaptations that have allowed multiple highly virulent clonal strains to emerge from within CC8 over the past 50 years.

## Abstract (precis)

In this work, we have removed some of the confusion surrounding the use of the name “USA500,” placed USA500 strains in the context of the CC8 group, and developed a strategy for assignment to subclades based on genome sequence. Our new phylogeny of USA300/USA500 will be a reference point for understanding the genetic adaptations that have allowed multiple highly virulent clonal strains to emerge from within CC8 over the past 50 years.

## INTRODUCTION

The name “USA500” is used to describe a group of methicillin-resistant Staphylococcus aureus (MRSA) clones that have emerged over the past 20 years as frequent causes of community-associated (CA) and health-care-associated infections in North America. USA500 was first defined as a distinct pulsed-field gel electrophoresis (PFGE) type (1). Like the better known USA300 PFGE type, which has caused an epidemic of community-acquired infections in the United States (2, 3), USA500 strains mostly have the multilocus sequence type (MLST) ST8 genotype and are part of the CC8 clonal complex (4). Both USA300 and USA500 carry the type IV SCC*mec* cassette conferring resistance to β-lactam antibiotics and have conserved mutations in their capsule locus (5). USA300 strains are distinguished from USA500 by having Panton-Valentin leukocidin (PVL) toxin genes within a prophage of the phiSA2 family (6). Isolates of the North American epidemic (NAE) USA300 lineage have an arginine catabolic mobile element (ACME) cassette next to SCC*mec*, whereas those of the South American epidemic (SAE) lineage have a copper and mercury resistance (COMER) element at same locus (7). USA300 strains also have a SaPI5 pathogenicity island containing *sek* and *seq* enterotoxin genes (8). The arginine deiminase (*arc*) and polyamine resistance (*speG*) genes on the ACME cassette and the PVL toxin have been proposed as key determinants of the enhanced ability to cause skin and soft tissue infections (SSTIs) and transmissibility of USA300 (3, 9), although strains with deletions in these genes have been frequently reported (2, 10, 11).

It has been postulated that USA300 evolved as a clonal lineage from within a background of USA500 strains (2, 12). However, genome sequencing studies suggested the relationship was more complex, with USA500 strains assigned to different clades within CC8 (2, 3, 5, 13). Nomenclature for USA500 strains is also complicated. USA500 strains collected at the CDC (Centers for Disease Control and Prevention) were subdivided into two groups based on closely related PFGE types: true “USA500” and “Iberian.” Before 2012, the assignment was based largely on PFGE. From 2012 onward, an algorithm for inferring USA500 and Iberian was implemented (https://www.cdc.gov/HAI/settings/lab/CCalgorithm.html), which combined PFGE, *spa*, MLST, and PCR amplicon-based detection of key horizontally acquired staphylococcal enterotoxin A (*sea*) and B (*seb*) genes (12). Confusingly, the term “Iberian” was also earlier used to describe a PFGE type from an MRSA epidemic in Spain and other countries between 1990 and 1995 that was found to be ST247 (CC8) with SCC*mec* type Ia (14–16).

The CDC’s Emerging Infections Program (EIP) conducts active, laboratory- and population-based surveillance for invasive MRSA infections (17). Strains typed as USA500/Iberian have represented a significant proportion of EIP MRSA isolates. In 2013 (the final year in which we drew collected strains for sequencing in this study), they constituted 13.5% of health care-associated MRSA (HA-MRSA) strains collected at 5 surveillance sites (https://www.cdc.gov/abcs/reports-findings/surv-reports.html). Notably, in Georgia the incidence was higher than other sites, with USA500/Iberian representing 20% of all HA-MRSA infections over the period from 2012 through 2015 (R. Overton, personal communication). While numerous projects have investigated USA300 evolution through comparative genomics (2, 3, 10, 11, 13), fewer genomic studies have been performed on USA500. Here we aimed to investigate the diversity of USA500 isolates causing invasive infections in the United States through analysis of a large set of strains collected through EIP surveillance. The goal of this work was to arrive at a genetic definition of USA500 that can be used for future typing efforts and to understand its relationship to USA300.

## RESULTS

### CC8 strains typed as “USA500/Iberian” fall into three major clades.

**FIG 1:**
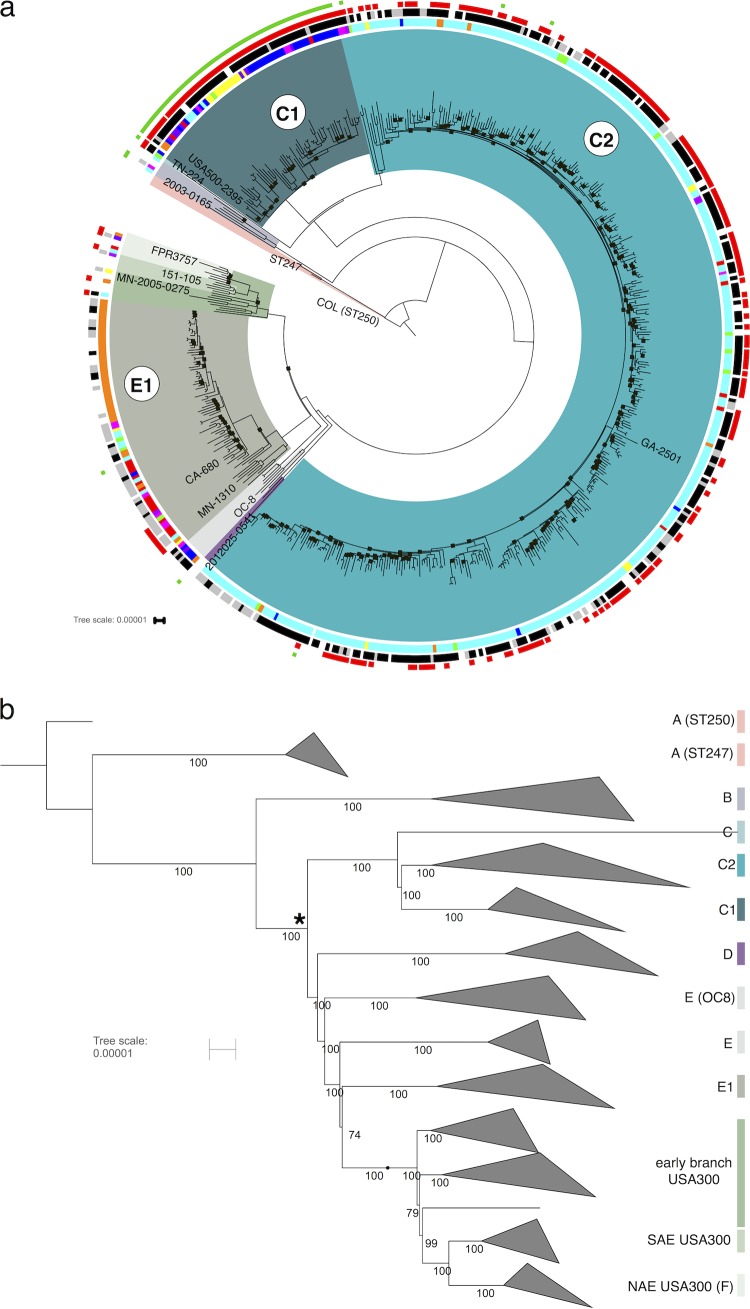
Maximum likelihood phylogeny of USA500 and other CC8 strains. The COL reference genome (68) was an outgroup. The major clades are color coded: F, green; E1, gray; D, purple; C1, dark blue; C2, blue; BA, brick red. The reference genome was strain 2395 in the C1 clade. The likelihood score for the tree was −3,374,456. (a) Circular view with locations of representative strains from each clade indicated in the text. Small black squares on the tree indicate branches supported by fewer than 90% bootstrap replicates. Outer ring 1 shows the results of inferred PFGE typing by PCR. Black indicates Iberian, gray indicates USA500, and white indicates other result or not done. The second ring is colored by U.S. state of origin: red, California; purple, Colorado; dark blue, Connecticut; light blue, Georgia; dark purple, Maryland; orange, Minnesota; yellow, New York; magenta, Oregon; green, Tennessee. The third ring (red squares) shows presence of an SaPI3/5-like site-specific integrase gene. The fourth ring (green squares) shows the presence of at least one copy of IS*256* in the genome. Only results from strains sequenced in this study are shown on the outer rings. The figure was created by iTOL (64). The tree with full metadata is publicly available at http://itol.embl.de/tree/1701401041445011519064958. Panel b is the same tree as panel a but with all multistrain clades collapsed. The internal node that is the common ancestor of all USA500/USA300 isolates is marked with an asterisk. The percentage of bootstrap support is shown for each of the branches. This tree is publicly available at http://itol.embl.de/tree/17014010416011519065058.

*De novo*-assembled contigs of the 539 strains typed as MRSA USA500, along with data from 24 published CC8 strains (listed with citations in Table S1 in the supplemental material), were aligned against the reference genome of strain 2395 (2,995,646 bp with a large plasmid [pUSA500] of 32,406 bp) (18). The 2395 strain, recovered from a wound infection in New York (18, 19), was originally assigned to the “Iberian” subgroup of USA500 based on the presence of the *sea* and *seb* genes. The whole-genome alignment of all 539 CC8 strains sequenced in this study plus 24 published genomes had a core region of 1,995 kbp (67.5% of the 2,956-kbp chromosome). Regions not part of the core alignment on the chromosome included prophages, pathogenicity islands, transposons, and other repeat sequences. Plasmid content was variable between strains and therefore excluded from consideration in the phylogenetic reconstruction. After recombinant regions were identified and removed, the final alignment consisting of 13,765 chromosomal single nucleotide polymorphisms (SNPs) was used to estimate a maximum likelihood tree (Fig. 1). We labeled the CC8 sublineages A to F using a scheme developed recently (20) (Fig. 1).

10.1128/mSphere.00571-17.9 TABLE S1  Samples included from external studies. Download TABLE S1, DOCX file, 0.1 MB. Copyright © 2018 Frisch et al. 2018 Frisch et al. This content is distributed under the terms of the Creative Commons Attribution 4.0 International license.

The phylogeny revealed that the majority of USA500/Iberian strains were in three major and discrete clades within CC8. Two of the USA500 clades were in the C sublineage and were designated C1 and C2. The other was in the E sublineage and was designated E1. Nineteen strains were placed outside these three clades: in USA300 (sublineage F), sublineage B, or deep-branching sublineages E and C (Fig. 1).

Metadata for each strain included patient age, epidemiological classification of infection (community-associated [CA], health care onset [HO], health care-associated community onset [HACO], or unknown), culture source (blood, cerebrospinal fluid [CSF], bone, etc.), outcome (lived, died, or unknown), and U.S. state of isolation. Using a permutation test, the three major USA500 clades defined here (C1, C2, and E1) were found to have a significantly nonrandom distribution in only the U.S. state of isolation metadata variable (*P* value of 9.7e−12), reflecting the geographical structuring of the USA500 clades.

**FIG 2:**
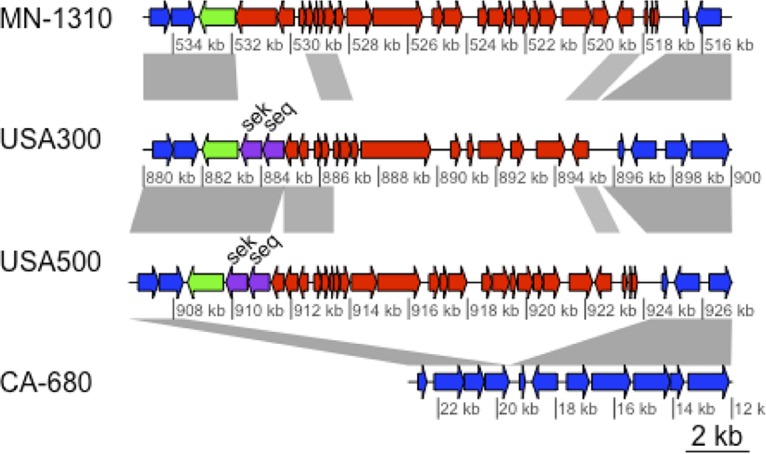
Divergent SaPI3/SaPI5 pathogenicity islands. The alignment shows an E1 strain (MN-1310) containing a novel SaPI3-E1 region, USA300-FPR375 and (SaPI5), USA500-2395 (SaPI3) and an example of an E1 strain lacking an inserted island at this locus (CA-680). Conserved chromosome genes are colored blue. SaPI3-E1 and SaPI share little and SaPI3 and SaPI3-E1 themselves share limited nucleotide similarity. The SaPI site-specific integrase gene is colored green, and the sek/seq enterotoxins are colored purple. Gray shading shows regions with >95% nucleotide identity in blastn alignments. The figure was created using genoplotR (69).

The C1 clade (64 strains from this study) contained the strain 2395 USA500 isolate that was used as the reference genome sequence for this study (18). The majority (52/64 [81%]) of samples were from Maryland, Connecticut, or New York. Most C1 strains were ST8, with minority populations of ST609 and ST1508. The C2 clade corresponded to the group labeled as “USA500-like” by Jamrozy et al. (13). These strains were predominantly from Georgia and Tennessee (351/378). One strain was typed as ST476, and the rest were ST8. E1 clade strains were predominantly isolated in California, Colorado, Minnesota, and Oregon (63/78 [81%]). This clade contained the BD02-25 strain originally used as the USA500 reference isolate (12). Of these, 36/78 (46%) were ST2253 and the rest were ST8. Two strains closely related to USA300 contained the S. aureus pathogenicity island (SaPI5) with USA300-like *seq* and *sek* genes but lacked the typical USA300 mobile elements ACME and PVL (Fig. 2).

**FIG 3:**
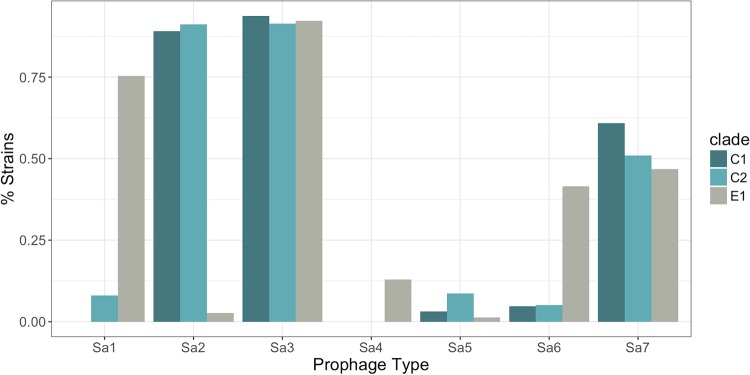
Frequency of prophages in the major USA500 subclades.

Seventy-six percent (39/51) of E1 strains were typed as “USA500” by the CDC algorithm, whereas 83% (304/368) of strains in C1 and C2 were typed as “Iberian” (Table 1). If we assume that E1 corresponded to the “USA500” inferred PFGE type and that the C1 and C2 strains were “Iberian” (because the majority of strains from each respective clade had these types), the number of correctly typed strains was 343 out of 419 (82% accuracy). The major reason for the relatively low accuracy was the frequent turnover of pathogenicity islands and prophages in USA500 genomes. The *seb* gene used in the CDC algorithm (in addition to *sek* and *seq*) was on the SaPI5 pathogenicity island in USA300; an analogous island in COL and USA500 (SaPI3) carried *sek* and *seq* (Fig. 2) (21). The SaPI was common in C1 and C2, but most E1 strains did not contain either SaPI3 or SaPI5, although a subclade of 8 strains was found to have a previously undescribed variant of SaPI3 at the same chromosomal locus (called here SaPI3-E1), with low sequence identity to the other islands and lacking *sek*/*seq* homologs (Fig. 2). Families of S. aureus prophages inserted into different conserved sites in the genome and varied in frequency of occurrence between strain groups (22). The *sea* gene used for typing, as well as *sak*, was usually found on the phiSA3 prophage. This element was inserted at a site in the *hlb* hemolysin B gene in C1/C2, but in the E1 strains the DNA sequence of the element was missing the *sea*/*sak* genes (Fig. 2). Most C1 and C2 strains carried the phiSA2 prophages, which were rare in E1. Conversely, phiSA1 and phiSA6 were more common in E1 (75% and 45%, respectively) than other clades (Fig. 3).

**TABLE 1:** Inferred PFGE type of strains in this study, by clade^a^

Clade	No. of strains sequenced		
	Sequenced	Inferred “USA500”	Inferred “Iberian”
C1	64	3	57
C2	378	61	247
E1	78	39	12
Other	19	8	7
Total	539	111	323

^a^ The strains sequenced in this study were placed in clades by their position on the phylogenetic tree (Fig. 1). The third and fourth columns are the inferred PFGE type to which the strains were assigned using methods described in https://www.cdc.gov/abcs/reports-findings/survreports/mrsa13.html. (Note that not all strains sequenced in this study were tested by the algorithm.)

### Emergence of the USA500 clades.

Initial analysis suggested that there was a clock-like structure to the phylogeny, with strains sampled in earlier years nearer the root than those from later years. Therefore, we attempted to estimate the dates of diversification of the USA500 clades using a Bayesian molecular clock method implemented by the BEAST2 software package (23) (see Fig. S1 in the supplemental material). Key parameter predictions that could be cross-referenced against other studies were consistent with our estimates. For instance, our estimate of the substitution rate parameter (1.158e−6 mutations per nucleotide site per year) was similar to those of previous S. aureus studies: e.g., 1.3e−6 (24), 1.53e−6 (11), 1.25e−6 (10), and 1.34e−6 (2) (with the caveats that some of these studies had partially overlapping data sets and each used slightly different evolutionary models). Furthermore, the estimate of 1989 as the time to the most recent common ancestor (tMRCA) of the NAE group USA300 strain was within the ranges of other recent publications (3, 10, 11). The C1, C2, E1, and F clades all appeared to have emerged at time points toward the middle of the 20th century. We noted that the C2 clade tMRCA estimation was likely significantly retarded by the three deeply branching isolates. When these were removed, the tMRCA was 1972 (1945 to 1992, 95% high posterior density intervals). Each of the major clades’ Tajima’s *D* statistics was negative (Table 2), consistent with a recent population size expansion scenario.

10.1128/mSphere.00571-17.1 FIG S1  Dated reconstruction of USA500 clades using BEAST 2. A log-normal relaxed molecular clock was employed (see Materials and Methods for details), and we used the dates of isolation to calibrate the clock. Color coding is as follows: light blue clade, C1; dark blue clade, C2; red, clade USA300; and green, clade E1. The scale axis gives the years from 2013 going backwards in time. Download FIG S1, DOCX file, 0.4 MB. Copyright © 2018 Frisch et al. 2018 Frisch et al. This content is distributed under the terms of the Creative Commons Attribution 4.0 International license.

**TABLE 2:** Time to the most recent common ancestor (tMRCA) for the main clades

Clade	Median estimated tMRCA^a^	Tajima’s *D*
C1	1937 (1873−1973)	−2.32
C2	1945 (1890–1978)	−2.63
E1	1950 (1899–1980)	−2.42
F	1951 (1901–1981)^b^	−1.90
F (NAE)	1989 (1976–1999)	−0.98
F (SAE)	1994 (1983–2001)	−0.85
USA300/USA500 (C–F)	1892 (1820–1952)	−2.33

^a^ In parentheses are the 95% highest posterior density intervals.

^b^ See reference 11 for details.

### USA500 clades differ in proportion of strains resistant to antibiotics.

**FIG 4:**
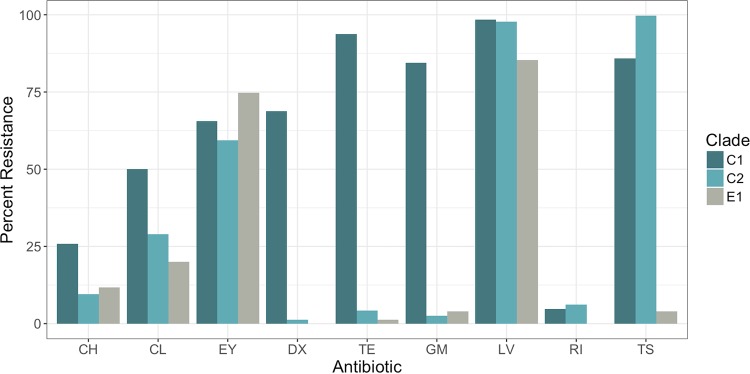
Percentage of resistance to antibiotics that showed significant variability between USA500 clades measured by rBMD. CH, chloramphenicol; CL, clindamycin; EY, erythromycin; DX, doxycycline; TE, tetracycline; GM, gentamicin; LV, levofloxacin; RI, rifampin; TS, trimethoprim/sulfamethoxazole.

The phenotypic antibiotic resistance profile was determined for most strains sequenced in this study using the reference broth microdilution (rBMD) method with CLSI (Clinical and Laboratory Standards Institute)-recommended interpretive criteria (25) (Fig. 4). There was no trend toward increased resistance to greater numbers of drugs per strain over the time period 2005 through 2013 (see Fig. S2a in the supplemental material). However, it was notable that C1 strains were resistant to a greater number of antibiotics than C2 and E1 strains (Fig. S2b). Almost all C1 strains were resistant to tetracycline (60/64) and gentamicin (54/64) (Fig. 4). Most C1 (55/64) and C2 (324/325) strains tested were also resistant to trimethoprim-sulfamethoxazole, a drug often used in treating community-acquired S. aureus SSTIs, but resistance was uncommon among E1 strains (3/72). These and other more sporadic resistance phenotypes were associated with the presence of horizontally acquired plasmids, the Tn*916* conjugative transposon (which conferred tetracycline resistance), or a 3.3-kb insertion element containing the trimethoprim resistance gene *dfrG* (13, 26–28).

10.1128/mSphere.00571-17.2 FIG S2  Antibiotic resistance by year. (a) All strains. (b) By USA500 clade. Download FIG S2, DOCX file, 0.2 MB. Copyright © 2018 Frisch et al. 2018 Frisch et al. This content is distributed under the terms of the Creative Commons Attribution 4.0 International license.

Most isolates tested (435/464 [94%]) were resistant to the fluoroquinolone (FQ) levofloxacin. We observed that FQ-susceptible strains tended to be on early branching lineages of the C2 and E1 clades. This mirrored the pattern seen in USA300, where a subgroup of strains that branched after an estimated point in 1994 were found to be FQ resistant (10, 11). These results suggested that resistance to FQ independently evolved in multiple emergent CC8 lineages.

### Conserved IS*256* insertion sites in C1 strains suggest expansion from an ancestor with multiple transposon insertions.

Insertion sequence IS*256* was previously shown to play a significant role in the hypertoxicity of strain 2395 and other isolates from the C1 clade (18). The genome was notable for the presence of 18 identical copies of the insertion sequence IS*256* (16 on the chromosome, 2 on the plasmid). Two IS*256* elements in inverted orientation flank gentamicin and trimethoprim resistance genes to form transposon Tn*4001*, which is part of the pUSA500 plasmid. One IS*256* element in the promoter of the *rot* (repression of toxin) gene, a master positive transcriptional regulator of toxin expression, was found to be responsible for increased cytotoxin production. The 73 USA500 isolates that contained at least one copy of IS*256* included all 64 in C1. Benson et al. speculated that the pUSA500 plasmid spread IS*256* to the USA500 chromosome (18). We found that 40 strains contained sequences similar to the pUSA500 plasmid, judged as having a BLAST match of more than 97% sequence identity over >13 kb of the 32-kb plasmid (accession no. CP007500.1). Only one strain carrying a pUSA500-like plasmid was outside the C1 clade (in C2). Meanwhile, seven strains outside C1 without a USA500-like plasmid had an IS*256*. Thus, there was not an absolute correlation of the presence of the plasmid with the transposon. Possibly the plasmid originally introduced the transposon in these strains and was cured after a time sufficient for IS*256* to transpose into the chromosome.

We determined the chromosomal locations of IS*256* insertions in our sequenced strains relative to the 2395 reference (18) in using the ISMapper tool (29). Within the C1 clade, the number of IS*256* chromosomal insertion locations ranged from 9 to 42, while outside C1, the range was 1 to 3 locations. There was no trend toward increase in number of insertions per isolate during the period of collection (2005 to 2013) (see Fig. S3a in the supplemental material). Eleven insertion sites were common among almost all C1 strains (Fig. S3b), suggesting that these were present in the last common ancestor of the clade. These 11 sites included one upstream of the *rot* gene (18).

10.1128/mSphere.00571-17.3 FIG S3  IS*256* elements. (a) Number of IS*256* elements in C1 genomes over time. (b) Sites for insertion of IS*256* on the 2395 chromosome. Download FIG S3, DOCX file, 0.1 MB. Copyright © 2018 Frisch et al. 2018 Frisch et al. This content is distributed under the terms of the Creative Commons Attribution 4.0 International license.

### USA500 C clade strains have a frameshift mutation in the *adsA* adenosine synthase gene

We noted that many USA500 strains had a premature stop in *adsA*, encoding adenosine synthase (previously called *sasH* [30]). The cell-wall-anchored protein encoded by *adsA* had been shown to aid in evasion of phagocytic clearance in blood, by catalyzing the production of adenosine, an anti-inflammatory signal molecule, from AMP (31). The wild-type AdsA protein has an LPXTG motif necessary for sortase-mediated anchoring to peptidoglycan on the cell surface (32) in the C terminus of the 773-amino-acid preprotein. The most important residues for 5′-nucleotidase activity are toward the N terminus: aspartic acid 127 and histidine 196 (see Fig. S4 in the supplemental material). All isolates in the C clade had a duplication of the “TCAA” quadruplet at nucleotide positions 340 to 343 of the wild type (see Fig. S5 in the supplemental material). The frameshift resulted in a truncated protein of 131 amino acids (as well as a predicted C-terminal stub of 636 amino acids) instead of the full-length 773-amino-acid sequence. The C-terminal stub was not predicted to have a signal sequence necessary for translocation out of the cytoplasm. Therefore, the truncated proteins, if they were stably expressed, would be predicted to be diminished in activity as surface-exposed adenosine synthases. This mutation clearly did not abolish the potential to cause human systemic illness, as might have been predicted from the result of earlier mouse bacteremia model studies (31), since all isolates were associated with invasive disease.

10.1128/mSphere.00571-17.4 FIG S4  Schematic of the *adsA* frameshift mutation. Shown are the approximate locations of the frameshift in the DNA sequence and important amino acid domains (red). The D127 and H196 were shown to reduce 5′-nucleosidase activity when substituted for with alanines. Download FIG S4, DOCX file, 0.1 MB. Copyright © 2018 Frisch et al. 2018 Frisch et al. This content is distributed under the terms of the Creative Commons Attribution 4.0 International license.

10.1128/mSphere.00571-17.5 FIG S5  Phylogenetic distribution of the *adsA* frameshift mutation. Strains with a frameshift are shaded light blue on the outer ring; wild-type strains are dark blue. Clade colors are the same as in Fig. 1. Only strains sequenced in this study are marked. Download FIG S5, DOCX file, 1.9 MB. Copyright © 2018 Frisch et al. 2018 Frisch et al. This content is distributed under the terms of the Creative Commons Attribution 4.0 International license.

## DISCUSSION

It is important to establish a consistent use of the name “USA500” that can be used for future epidemiological studies and comparisons. The terms “USA500” and “USA300” are derived from a PFGE typing scheme established in early 2000s (33) and represent different lineages within a single clonal complex (CC8) that acquired SCC*mec* type IV cassettes and increased in incidence in the human population. In essence, three definitions of the USA500 strain have been used in the literature, as described below: (i) USA500 PFGE type, (ii) USA500 *sensu stricto* as a single clade within CC8, and (iii) USA500 *sensu lato* as the genetic background to USA300 (e.g., as proposed by Glaser et al. [2]).

### (i) USA500 PFGE type.

USA500 is the original designation associated with MRSA strain types in the United States, based on PFGE (1). However, PFGE will be performed less frequently on future isolates as clinical genome sequencing becomes more routine (34). We also showed that PFGE (before 2012) and the algorithm (after 2012) for inferring USA500 from “Iberian” PFGE using MLST or *spa* and *sea*/*seb* PCR was only 82% accurate in distinguishing USA500 and Iberian USA500 pulse types (Fig. 1; Table 1).

### (ii) USA500 *sensu stricto* as a single clade within CC8.

If USA500 was represented a single clade, the primary candidates would be C1 and C2 or E1. Strain 2395 (C1) has been the reference genome for USA500 since the publication by Benson et al. (15). This strain was assumed to be representative of a unitary USA500 clade in the recent genomic analysis of CC8 by Strauß et al. (34), which led the authors to conclude that USA500 was not a direct ancestor of USA300. Alternatively, the marker-based typing scheme proposed by Li et al. designated the E1 clade as USA500 (11) (Fig. 1; Table 1). Designation of one or the other of these clades as the sole USA500 clade would have the advantage of casting USA500 as a true monophyletic clade. The problem is that any *sensu stricto* definition excludes many other strains that are commonly referred to in the literature as USA500.

### (iii) USA500 *sensu lato* as the genetic background to USA300.

As proposed by Glaser et al. (2), USA500 *sensu lato* could be considered the genetic background to USA300. All CC8 non-USA300 strains that derive from an ancestor that contained a signature *cap5D* A nucleotide insertion at position 994 in the gene (5) could be considered USA500. Based on this definition, the CC8 sublineages C, D, and E (20) would all be considered USA500 (Fig. 1). We believe the “*sensu lato*” definition has the advantages of being simple and inclusive. One consequence of using this definition is that it would sequester some strains not previously considered USA500, including the recently described epidemic Russian clone OC8 (35) (Fig. 1). The clade containing all descendants of the common ancestor of USA300 and USA500 isolates (subgroups C to F) would be called USA300/USA500 (*sensu lato*).

The whole-genome phylogeny and molecular clock analysis revealed that the three major USA500 clades collected by the EIP had undergone population expansions in the United States from around the middle of the 20th century. USA300 emerged from the F sublineage and spread within the United States and internationally (36) rather than remaining regionally concentrated, as the USA500 clades did. Including the recent Russian OC8 strain (35), we now know there have been at least five significant expansions of virulent strains carrying SCC*mec* type IV cassettes from within the CC8 clonal complex (USA300, OC8, and the 3 USA500 clades). CC8 was also the origin of the first MRSA strains with the type I SCC*mec* cassette (ST250) in the early 1960s (37, 38). It has been suggested that the CC8 background in general and USA500/USA300 in particular have intrinsic high virulence potential (39, 40). Li et al. showed that USA300 strains and USA500 (clade E1) strain BD02-25 had greater virulence in a bacteremic mouse model than other CC8 strains and enhanced resistance to human antimicrobial peptides (12). Benson et al. also demonstrated the unusually high toxin levels of the C1 strain 2395 (18).

Each of the five expansions within CC8 (USA300, OC8, C1, C2, and E1) may have been the result of either specific genetic adaptations or chance events or a combination of both. Interestingly, there are parallels in the types of genetic changes acquired by each strain expansion and also unique differences. Antibiotic resistance may have played a role in the expansion of the C1 clade in particular (Fig. 4). Fluoroquinolone resistance, as an example, likely evolved in parallel on at least 4 occasions by the strains that form part of the study. There must have been particular selection pressure for survival of the effects of this class of antibiotics (possibly administered to treat other infections), and the mutations probably have low fitness cost, allowing them to persist in the population even in the absence of an antibiotic selective pressure. It has been suggested that the propensity for secretion of fluoroquinolones onto the skin through sweat may lead to high enough drug concentrations to effect selection (41).

In the case of OC8 and C1, parallel acquisition of IS*256* seemed to be associated with expansion (18, 35). An IS*256* element in strain 2395 (clade C1) disrupted the *rot* (repressor of toxicity) locus and increased toxin production (18). C1 strains containing IS*256* were found to be more cytotoxic for human neutrophils (18, 42) and also exhibited greater virulence for mice in a systemic infection model (43). The OC8 strain had 19 IS*256* copies, two of which, in inverted orientation, facilitated a 1-Mb genomic inversion in the main chromosome (35). IS*256* is a catalyst for expansion in diverse S. aureus lineages and may affect numerous other phenotypes—for example, vancomycin resistance (44). IS*256* transposition activity is enhanced by antibiotic concentration, which may be a clue in understanding the recent spread of the element in S. aureus and other pathogens (45–47). We surveyed a database of 3,755 published S. aureus Illumina genome assemblies for the IS*256* transposase, finding identical or nearly identical (a maximum 2-nucleotide [nt] difference) sequences in a number of recent clonally expanding genotypes. These included 41/41 ST772 strains, an emerging Indian strain, CA-MRSA (48), and 50/50 ST239, a worldwide HA-MRSA clone contained IS*256*. IS*256* was common (37/262 strains) in ST398, associated with human/livestock transmission (49). No USA300 strain contained IS*256*. The fact that very similar IS*256* elements can move between S. aureus clonal complexes is fascinating given the known genetic barriers to transfer (50, 51). Genes with 100% nucleotide identity to the USA500 IS*256* transposase gene were also found by BLAST in other bacterial species. These species included Staphylococcus epidermidis, Staphylococcus haemolyticus, Staphylococcus pseudintermedius, Staphylococcus warneri, Staphylococcus capitis, Enterococcus faecium, Enterococcus faecalis, Enterococcus durans, Clostridiales bacterium, Clostridium difficile, Mycoplasma mycoides, Pseudomonas aeruginosa, and Escherichia coli. This suggested IS*256* is part of a recent genetic exchange community (26) that encompassed diverse genetic groups within S. aureus as well as several other pathogen species.

For USA300, the mobile ACME (or COMER) elements, *speG*, and PVL toxins were likely important for its success in community-associated infection (40). The E1 USA500 clade is most closely related to USA300 and may share some of its yet not fully understood adaptations that promote enhanced expression of extracellular toxins and increased transmission rate. The C subgroup had a synapomorphic frameshift mutation in the *adsA* gene encoding the core (52) surface protein. Previous studies have shown that AdsA is required for full virulence in mouse bacteremia models. A possible explanation invoking pathoadaptation is that the *adsA* functions to reduce inflammation when S. aureus is on the skin by promoting production of adenosine, a purine nucleotide and antagonist (53). Inflammation may contribute to more frequent transmission and spread of the bacteria by causing rashes and skin damage. The finding that the disrupted *adsA* gene is common in clinical USA500 MRSA isolates from the United States may also be significant in the future given that the encoded surface protein is a potential vaccine target (54).

In conclusion, whole-genome sequencing has resolved the conundrum of USA500 nomenclature and unveiled possibly important genetic changes (SNPs and horizontal acquisition of genes) that played a role in evolution of pathogenic CC8 MRSA by promoting virulence and/or transmission. These mutations can be used for subtyping CC8 strains using PCR or genome-based types. Functional studies are needed now to disentangle which mutations enhance the success of community and hospital pathogens and which are just random evolutionary noise.

## MATERIALS AND METHODS

### Bacterial strains.

Invasive MRSA isolates were collected from California (CA), Colorado (CO), Connecticut (CT), Georgia (GA), Maryland (MD), Minnesota (MN), New York (NY), Oregon (OR), and Tennessee (TN) as part of the Emerging Infections Program (EIP) of the Centers for Disease Control and Prevention (CDC) as previously described (55). All isolates were characterized at the CDC by SCC*mec* typing, detection of staphylococcal toxins, antimicrobial susceptibility testing, pulsed-field gel electrophoresis (PFGE) typing, and PCR typing as previously described (1, 55). PFGE was only performed from 2005 to 2008; from 2009 to 2011, an algorithm (https://www.cdc.gov/HAI/settings/lab/inferred-PFGE-algorithm.html) was used to infer PFGE type, and then between 2012 and 2013, a second algorithm incorporating *spa* typing was used (https://www.cdc.gov/HAI/settings/lab/CCalgorithm.html). From 758 strains classified as USA500/Iberian, 549 strains were chosen for whole-genome sequencing based on maximizing geographic and genetic diversity, based on metadata collected at the time of isolation. We used all the isolates from states other than Georgia, which had the majority of isolates. Strains from Georgia were randomly down-sampled using the criterion that all sampling years, hospitals, and unique PFGE patterns would be represented in the set selected for sequencing. The number of isolates chosen from each year varied between a high of 74 (2005) to a low of 49 (2013) (see Fig. S6 in the supplemental material).

10.1128/mSphere.00571-17.6 FIG S6  Number of strains sequenced each year (2005 to 2013). Download FIG S6, DOCX file, 0.1 MB. Copyright © 2018 Frisch et al. 2018 Frisch et al. This content is distributed under the terms of the Creative Commons Attribution 4.0 International license.

### DNA isolation.

MRSA was grown overnight at 35°C on Trypticase soy agar with 5% sheep’s blood (BAP) (Becton, Dickinson and Company, Sparks, MD). Bacterial colonies from the third and fourth quadrants of the BAP were transferred into 1.5 ml of phosphate-buffered saline (PBS) with 0.02% Tween (PBST) and centrifuged at 13,200 × *g* for 2 min at room temperature. Cells were resuspended in 1.5 ml of PBST and centrifuged at 13,200 × *g* for 2 min at room temperature two more times and resuspended in 1.5 ml PBST. Nine hundred microliters of each sample was transferred into a Lysing Matrix E tube (MP Biochemicals), vortexed for 3 min, and then centrifuged at 5,000 × *g* for 1 min, and 200 µl was transferred into an SEV cartridge (MP Biochemicals) and processed per the manufacturer’s instructions on the Maxwell 16 or Maxwell 16 MDx instrument (Promega).

### Whole-genome shotgun sequencing.

Libraries constructed using whole-genome DNA preparations were sequenced using an Illumina HiSeq 2500 instrument. Raw read data were deposited in the NCBI Short Read Archive under project accession no. PRJNA316461. The median sequence coverage for each genome was 89-fold with a median per base *Q* score of 37. One strain was excluded due to library failure.

### Genome assembly and annotation.

Strains were assembled *de novo* using SPAdes v3.7.1 (56) and annotated using PROKKA v1.11 (57). FASTQ sequencing output files from strains with more than 100× coverage were down-sampled using a custom script (https://gist.github.com/rpetit3/9c623454758c9885bf81d269e3453b76) based on the seqtk toolkit (https://github.com/lh3/seqtk). Antibiotic resistance phenotypes were predicted for each strain based on the methods of Gordon et al. (58). Roary (59) was used to estimate a pan-genome from *de novo*-assembled contigs of the strains sequenced in the study. The MLST was ascertained using SRST2 (60). Two strains were excluded because pangenome content suggested they were not S. aureus, and 8 strains fell outside CC8 based on MLST patterns.

### BLAST comparisons to assembled genome sequences.

Blast+ v2.2.28 was used for alignments. For short nucleotide sequences (<31 nt), we used the blastn with the “blastn-short” task and called matches with a greater than 90% identity and an alignment length of at least 15. For alignment of protein sequences, we used the tblastn program and called matches with at least 97% identity. For most larger nucleotide alignments, we used blastn with the megablast task and called matches with greater than 97% identity. For antibiotic resistance genes, we followed the guidelines of Gordin et al. (58) and used blastn with a word size parameter of 17, gapopen of 5, and gapextend of 2 and saved matches where the identity multiple by the ratio of the hit to the total length of the gene was greater than 0.8 (or 0.3 in the case of the *blaZ*, *fusB*, and *far* genes).

### IS*256* insertion sites.

All strains found to contain IS*256* were processed using ISMapper v1.2 (29) to determine the transposon insertion sites. ISMapper used the paired-end FASTQ files of reads for each genome, the reference genome assembly (USA500-2395; accession no. CP007499.1) (18) and an IS*256* query sequence (accession no. NC_013321.1) (33).

### Phylogenetic tree estimation.

A whole-genome alignment of *de novo*-assembled contigs from 539 CC8 strains from this study and 24 CC8 strains described in other papers was processed using Parsnp (61). The alignment length was 2,361,133 bp. Potential recombination sites were identified using ClonalFrameML (24) based on a maximum likelihood (ML) guide tree constructed by PhyML (62, 63), removed using a custom R script, leaving a final alignment of 2,359,393 bp with 18,755 variable sites (SNPs). The alignment file, R script, and ClonalFrameML output listing recombinant sites have been made publicly available on FigShare (https://doi.org/10.6084/m9.figshare.5915257.v1). We performed ML tree estimation on the 2,359,393-bp alignment with 1,000 bootstraps using RAxML version 8.2.11 (63) with a GTRGAMMA model and one partition. The resulting phylogenetic trees were visualized with the Interactive Tree of Life (iTOL) web service (64). We chose ST250 COL (accession no. CP000046.1 [13]) as the outgroup, as it was the earliest branching strain in subgroups A to F when we included a more divergent ST630 in a pilot phylogeny. One strain in group F, CT-172, was pruned from the final tree because of a long branch. Visual inspection using the gingr tool (61) revealed the presence of a likely large recombinant region in this strain between 2.47 and 2.59 Mbp on the USA500-2395 reference coordinates that had not been detected by ClonalFrameML.

### Statistical tests of metadata.

Seven metadata categories for each sample were provided: state, hospital identification, year of collection, culture source, patient age, in-hospital patient mortality (deceased, alive, and unknown) and sample class (HACO, CO, HA, and CA). A case was classified as HO if the MRSA culture was obtained on or after the fourth calendar day of hospitalization (where admission was hospital day 1). A case was classified as HACO if the culture was obtained in an outpatient setting or before the fourth calendar day of hospitalization and had one or more of the following: (i) a history of hospitalization, surgery, dialysis, or residence in a long-term-care facility in the previous year, or (ii) the presence of a central vascular catheter (CVC) within 2 days prior to MRSA culture. Finally, a case was classified as community-associated (CA) if none of the previously mentioned criteria were met. To test the significance of these categories on which clade the sample belongs to, we ran a permutation test on each category, and adjusted the output *P* values with a Bonferroni correction. Tests were implemented using the Independence Test from the coin R package (65) and p.adjust from the base statistics R package.

### Molecular dating analysis.

We used BEAST 2.4.5 (23) to conduct the molecular dating analysis, incorporating a coalescent Bayesian skyline demographic model with 5 groups. We picked up to 5 isolates from each sampling year for each of the clades C1, C2, and E1. Where there were more than 5 strains in a clade for a given year, we randomly selected 5 using the R *sample* function. We picked all dated samples from sublineage F in Fig. 1 and representatives of ST247, ST250, and sublineages B and D as outgroups. COL (ST250) was set as the root of the tree as in the phylogenetic analysis described above. In total, 151 samples were used. We experimented with different subsampling strategies before selecting 130,000 randomly picked sites. We found larger data configurations (more isolates and/or more sites) did not reach effective sample sizes (ESSs) of >200 for many parameters even after 300,000,000 generations. We used a GTR+R substitution model with the correction for among-site variation. We obtained good estimates of the posterior distribution of the parameters in this analysis, as these parameters reached over 200 ESSs. We set an uncorrelated log-normal relaxed clock and calibrated the clock using the dates of collection of the isolates. The final analysis was run for 200,000,000 generations, sampling every 20,000 generations and discarding the first 20,000,000 generations as burn-in. We evaluated the convergence of the analysis by checking that all the parameters reached ESS values of >200 and by analyzing the trace plots of the likelihood scores. Furthermore, we ran the final analysis twice so as to check that the analysis was converging and the two runs reached very similar results (see Table S2 and Fig. S7 in the supplemental material). To test whether the subsampling strategy accurately reproduced variation in the data, given that we only used a subset of the sites, we created another 3 replicates (randomly subsampling the same number of sites), and molecular dating analyses were run on each replicate. We obtained similar results across all the replicates (see Fig. S8 in the supplemental material). Tajima’s *D* values for clades were calculated using VariScan (66).

10.1128/mSphere.00571-17.7 FIG S7  Convergence of molecular clock analyses. Marginal density of the tree likelihood for the two runs of the molecular clock analysis. The convergence of the two runs is clear from the overlap of the distributions. Download FIG S7, DOCX file, 0.2 MB. Copyright © 2018 Frisch et al. 2018 Frisch et al. This content is distributed under the terms of the Creative Commons Attribution 4.0 International license.

10.1128/mSphere.00571-17.8 FIG S8  Molecular clock replicate runs. Marginal posterior density of the tree height for 3 replicate runs (blue, orange, and red) and the final molecular clock analysis (black). The marginal densities of the three replicates extensively overlap that of the final analysis. Download FIG S8, DOCX file, 0.4 MB. Copyright © 2018 Frisch et al. 2018 Frisch et al. This content is distributed under the terms of the Creative Commons Attribution 4.0 International license.

10.1128/mSphere.00571-17.10 TABLE S2  Convergence of the two molecular clock analysis runs. The table shows similar estimates of the tMRCA for the three major clades in this study for each run. Download TABLE S2, DOCX file, 0.1 MB. Copyright © 2018 Frisch et al. 2018 Frisch et al. This content is distributed under the terms of the Creative Commons Attribution 4.0 International license.

### Clade attribution.

We used the VCF format output of the whole-genome alignment to identify 31-mers unique to each major USA500 clade and USA300. Using Jellyfish (67), samples were queried for a nonzero count of each 31-mer from this list. Assignment of a strain to a clade was based on the presence of a strain-specific 31-mer from only one clade and the absence of 31-mers from other clades. We also extracted 31-mers associated with canonical SNPs defined by Bowers et al. (20) and used these to assign strains to sublineages A to F.
