# Supplementary material for: Invasive Methicillin-Resistant Staphylococcus aureus USA500 Strains from the U.S. Emerging Infections Program Constitute Three Geographically Distinct Lineages
Source: mSphere. 2018 May 2;3(3):e00571-17. doi: 10.1128/mSphere.00571-17 (PMC5932375; doi:10.1128/mSphere.00571-17)
Supplement: TABLE S1 [file sph003182533st1.docx]

##### Supplemental Table 1 Samples included from external studies

| **Sample Name** | **Clade** | **Citation** |
| --- | --- | --- |
| 2395 | C1 | [(Benson et al. 2014)](https://paperpile.com/c/lIaDrO/qlUn) |
| COL | A (ST250) | [(Gill et al. 2005)](https://paperpile.com/c/lIaDrO/abBr) |
| CA12 | F (USA300 SAE) | [(Planet et al. 2015)](https://paperpile.com/c/lIaDrO/Esne) |
| HUV05 | F (USA300 ‘early branching’) | [(Planet et al. 2015)](https://paperpile.com/c/lIaDrO/Esne) |
| V2200 | F (USA300 ‘early branching’) | [(Planet et al. 2015)](https://paperpile.com/c/lIaDrO/Esne) |
| M121 | F (USA300 SAE) | [(Planet et al. 2015)](https://paperpile.com/c/lIaDrO/Esne) |
| 2003-0165 | B | (Bowers, et al mSphere, mSphere00157-17 submitted) |
| 20043-0165 | B | (Bowers, et al mSphere, mSphere00157-17 submitted) |
| 2012014-0233 | D | (Bowers, et al mSphere, mSphere00157-17 submitted) |
| 2012025-0541 | D | (Bowers, et al mSphere, mSphere00157-17 submitted) |
| AI-2012020-0222 | A (ST247) | (Bowers, et al mSphere, mSphere00157-17 submitted) |
| AI-2012019-0011 | A (ST247) | (Bowers, et al mSphere, mSphere00157-17 submitted) |
| NY-2012 | Outgroup (ST630)* | (Bowers, et al mSphere, mSphere00157-17 submitted) |
| MN-2011a | Outgroup (ST630)* | (Bowers, et al mSphere, mSphere00157-17 submitted) |
| 151-05 | F (USA300 SAE) | (Bowers, et al mSphere, mSphere00157-17 submitted) |
| MN1250 | F (USA300 SAE) | (Bowers, et al mSphere, mSphere00157-17 submitted) |
| PA-2011a | F (USA300 ‘early branching’) | (Bowers, et al mSphere, mSphere00157-17 submitted) |
| MN-2005-0275 | F (USA300 ‘early branching’) | (Bowers, et al mSphere, mSphere00157-17 submitted) |
| 2012007-0120 | F (USA300 ‘early branching’) | (Bowers, et al mSphere, mSphere00157-17 submitted) |
| PA-2009a | F (USA300 ‘early branching’) | (Bowers, et al mSphere, mSphere00157-17 submitted) |
| 2012046-0120 | F (USA300 ‘early branching’) | (Bowers, et al mSphere, mSphere00157-17 submitted) |
| 2012017-0045 | F (USA300 ‘early branching’) | (Bowers, et al mSphere, mSphere00157-17 submitted) |
| TCH1516 | F (USA300 NAE) | [(Highlander et al. 2007)](https://paperpile.com/c/lIaDrO/pa3Z) |
| FPR3757 | F (USA300 NAE) | [(Diep et al. 2008)](https://paperpile.com/c/lIaDrO/7Ir7) |

*Not shown on figure 1.

#####

##### Supplementary Table 2

Convergence of the two molecular clock analysis runs. The table shows similar estimates estimates of the tMRCA for the three major clades in this study for each run.

| Clade | First run | Second run |
| --- | --- | --- |
| C1 | 1936.6474 (1873.4643-1973.3851) | 1936.8894 (1877.3503 -1975.9442) |
| C2 | 1944.8372 (1890.4467-1978.5006) | 1944.3494 (1891.8237-1978.1858) |
| E1 | 1950.3324 (1898.8977-1980.0225) | 1950.5062 (1902.2744-1980.5834) |

Median estimates and 95% HPD interval in parentheses

[Benson, Meredith A., Elizabeth A. Ohneck, Chanelle Ryan, Francis Alonzo 3rd, Hannah Smith, Apurva Narechania, Sergios-Orestis Kolokotronis, et al. 2014. “Evolution of Hypervirulence by a MRSA Clone through Acquisition of a Transposable Element.” *Molecular Microbiology* 93 (4): 664–81. https://doi.org/](http://paperpile.com/b/lIaDrO/qlUn)[10.1111/mmi.12682](http://dx.doi.org/10.1111/mmi.12682)[.](http://paperpile.com/b/lIaDrO/qlUn)

[Diep, Binh An, Gregory G. Stone, Li Basuino, Christopher J. Graber, Alita Miller, Shelley-Ann des Etages, Alison Jones, et al. 2008. “The Arginine Catabolic Mobile Element and Staphylococcal Chromosomal Cassette Mec Linkage: Convergence of Virulence and Resistance in the USA300 Clone of Methicillin-Resistant Staphylococcus Aureus.” *The Journal of Infectious Diseases* 197 (11): 1523–30. https://doi.org/](http://paperpile.com/b/lIaDrO/7Ir7)[10.1086/587907](http://dx.doi.org/10.1086/587907)[.](http://paperpile.com/b/lIaDrO/7Ir7)

[Gill, Steven R., Derrick E. Fouts, Gordon L. Archer, Emmanuel F. Mongodin, Robert T. Deboy, Jacques Ravel, Ian T. Paulsen, et al. 2005. “Insights on Evolution of Virulence and Resistance from the Complete Genome Analysis of an Early Methicillin-Resistant Staphylococcus Aureus Strain and a Biofilm-Producing Methicillin-Resistant Staphylococcus Epidermidis Strain.” *Journal of Bacteriology* 187 (7): 2426–38. https://doi.org/](http://paperpile.com/b/lIaDrO/abBr)[10.1128/JB.187.7.2426-2438.2005](http://dx.doi.org/10.1128/JB.187.7.2426-2438.2005)[.](http://paperpile.com/b/lIaDrO/abBr)

[Highlander, Sarah K., Kristina G. Hultén, Xiang Qin, Huaiyang Jiang, Shailaja Yerrapragada, Edward O. Mason Jr, Yue Shang, et al. 2007. “Subtle Genetic Changes Enhance Virulence of Methicillin Resistant and Sensitive Staphylococcus Aureus.” *BMC Microbiology* 7 (November): 99. https://doi.org/](http://paperpile.com/b/lIaDrO/pa3Z)[10.1186/1471-2180-7-99](http://dx.doi.org/10.1186/1471-2180-7-99)[.](http://paperpile.com/b/lIaDrO/pa3Z)

[Inouye, Michael, Harriet Dashnow, Lesley-Ann Raven, Mark Schultz, Bernard Pope, Takehiro Tomita, Justin Zobel, and Kathryn Holt. 2014. “SRST2: Rapid Genomic Surveillance for Public Health and Hospital Microbiology Labs.” *Genome Med.* https://doi.org/](http://paperpile.com/b/lIaDrO/y91jY)[10.1186/s13073-014-0090-6](http://dx.doi.org/10.1186/s13073-014-0090-6)[.](http://paperpile.com/b/lIaDrO/y91jY)

[Marçais, Guillaume, and Carl Kingsford. 2011. “A Fast, Lock-Free Approach for Efficient Parallel Counting of Occurrences of K-Mers.” *Bioinformatics*  27 (6): 764–70. https://doi.org/](http://paperpile.com/b/lIaDrO/yHKLl)[10.1093/bioinformatics/btr011](http://dx.doi.org/10.1093/bioinformatics/btr011)[.](http://paperpile.com/b/lIaDrO/yHKLl)

[Planet, Paul J., Lorena Diaz, Sergios-Orestis Kolokotronis, Apurva Narechania, Jinnethe Reyes, Galen Xing, Sandra Rincon, et al. 2015. “Parallel Epidemics of Community-Associated Methicillin-Resistant Staphylococcus Aureus USA300 Infection in North and South America.” *The Journal of Infectious Diseases* 212 (12): 1874–82. https://doi.org/](http://paperpile.com/b/lIaDrO/Esne)[10.1093/infdis/jiv320](http://dx.doi.org/10.1093/infdis/jiv320)[.](http://paperpile.com/b/lIaDrO/Esne)
