# Supplementary material for: Invasive Methicillin-Resistant Staphylococcus aureus USA500 Strains from the U.S. Emerging Infections Program Constitute Three Geographically Distinct Lineages
Source: mSphere. 2018 May 2;3(3):e00571-17. doi: 10.1128/mSphere.00571-17 (PMC5932375; doi:10.1128/mSphere.00571-17)
Supplement: FIG S1 [file sph003182533sf1.docx]

##### Supplemental Figure 1. Dated reconstruction of USA500 clades using BEAST 2

A lognormal relaxed molecular was employed (see methods for details) and we used the dates of isolation to calibrate the clock. Colour-coding is as follows: light blue clade C1, dark blue clade C2, red clade USA300 and green called E1. The scale axis gives the years from 2013 going backwards in time.

#####

##### 
