# Supplementary material for: Invasive Methicillin-Resistant Staphylococcus aureus USA500 Strains from the U.S. Emerging Infections Program Constitute Three Geographically Distinct Lineages
Source: mSphere. 2018 May 2;3(3):e00571-17. doi: 10.1128/mSphere.00571-17 (PMC5932375; doi:10.1128/mSphere.00571-17)

##### Supplemental Figure 2a. Antibiotic resistances by year all strains

##
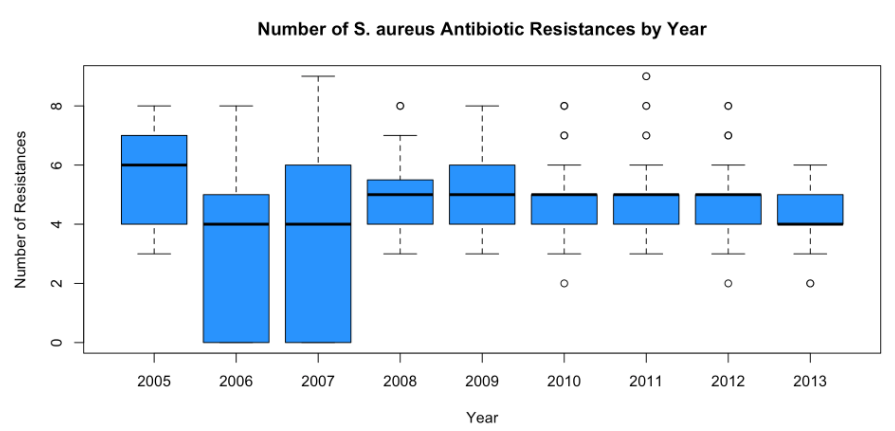


##

##

##### Supplemental Figure 2b. Antibiotic resistances by year by USA500 clade

##
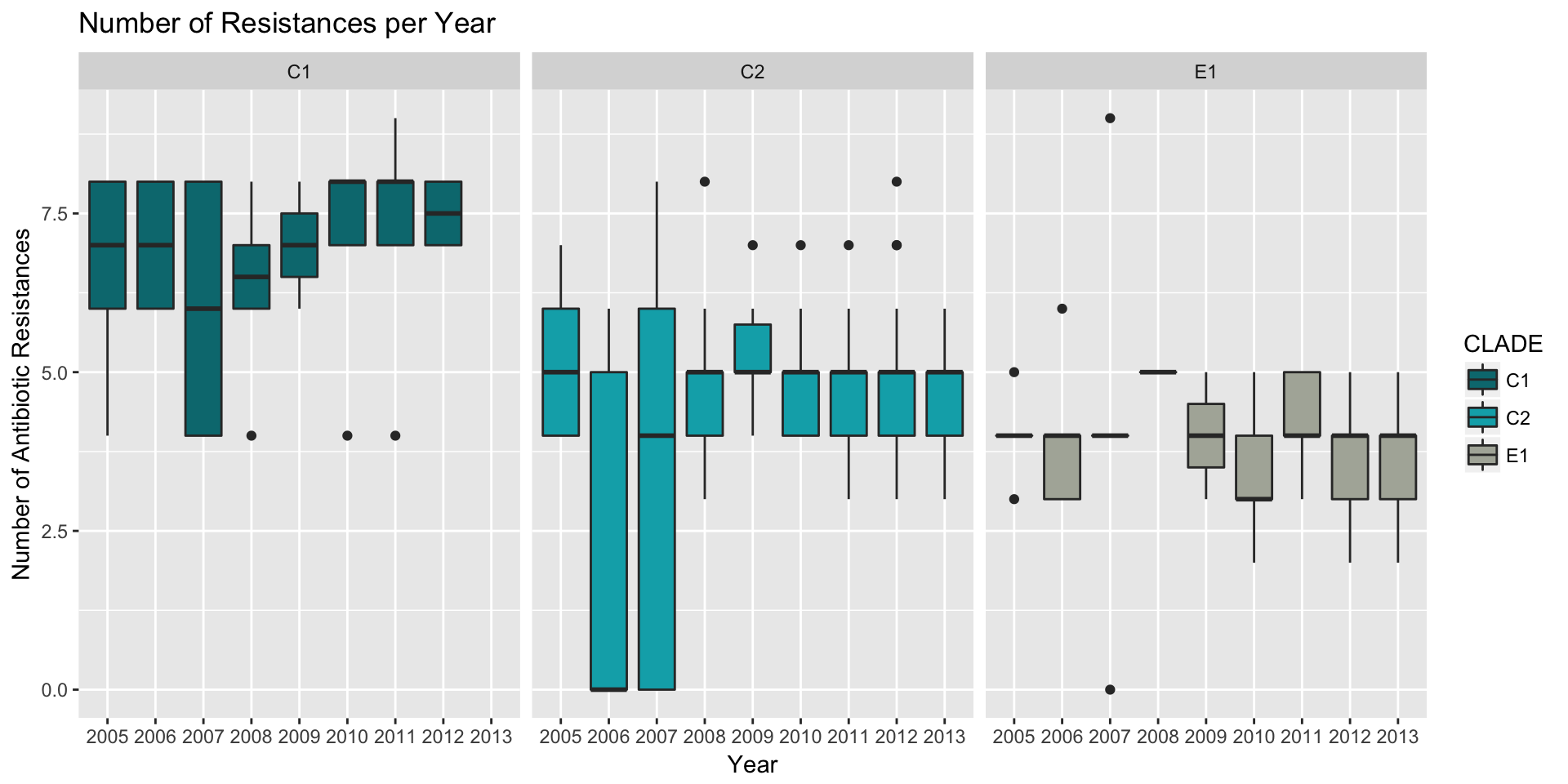

Supplement: FIG S2 [file sph003182533sf2.docx]
