# Supplementary material for: Invasive Methicillin-Resistant Staphylococcus aureus USA500 Strains from the U.S. Emerging Infections Program Constitute Three Geographically Distinct Lineages
Source: mSphere. 2018 May 2;3(3):e00571-17. doi: 10.1128/mSphere.00571-17 (PMC5932375; doi:10.1128/mSphere.00571-17)
Supplement: FIG S3 [file sph003182533sf3.docx]

##### Supplemental Figure 3a. Number of IS256 elements in C1 genomes over time


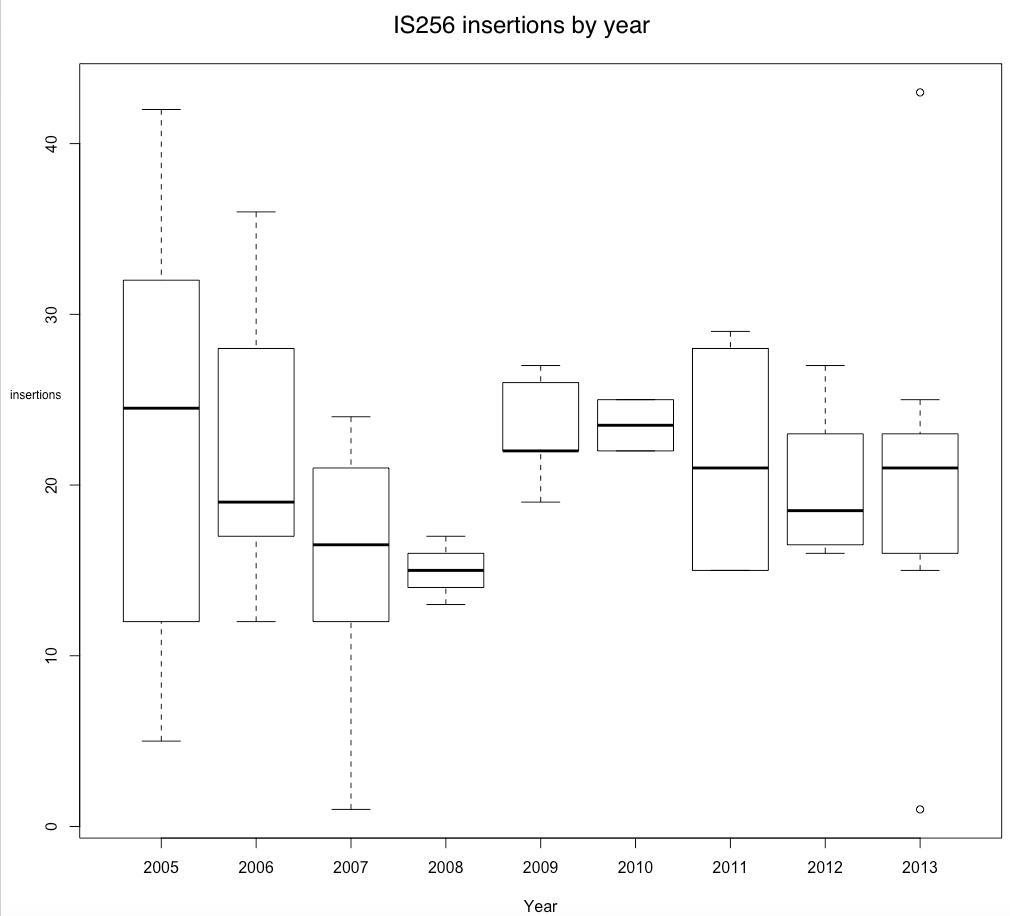


##### Supplemental Figure 3b. Sites for insertion of IS256 on the 2395 chromosome.


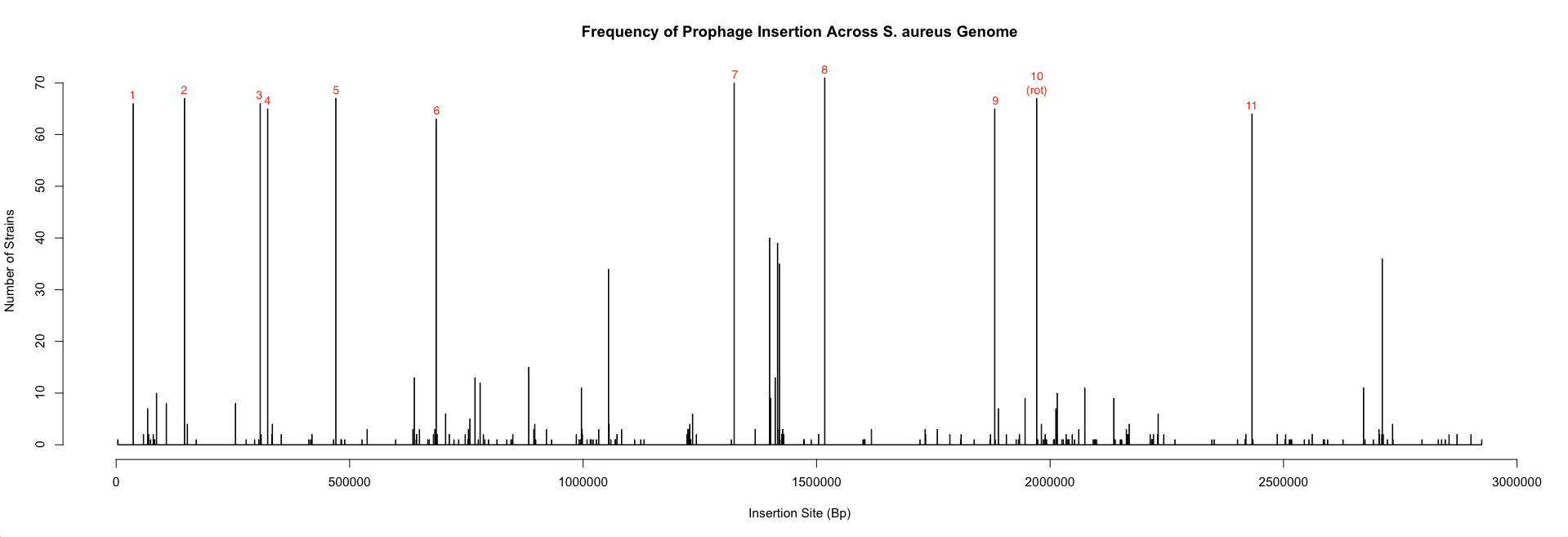


X-axis is the coordinate position on the MRSA 2395 chromosome.
