# Supplementary material for: Invasive Methicillin-Resistant Staphylococcus aureus USA500 Strains from the U.S. Emerging Infections Program Constitute Three Geographically Distinct Lineages
Source: mSphere. 2018 May 2;3(3):e00571-17. doi: 10.1128/mSphere.00571-17 (PMC5932375; doi:10.1128/mSphere.00571-17)
Supplement: FIG S4 [file sph003182533sf4.docx]

Supplemental Figure 4. Schematic of the *adsA* frameshift mutation.

L of the frameshift in the DNA sequence and important amino acid domains (red). The D127 and H196 were shown to reduce 5’-nucleosidase activity when substituted with alanines.

#####
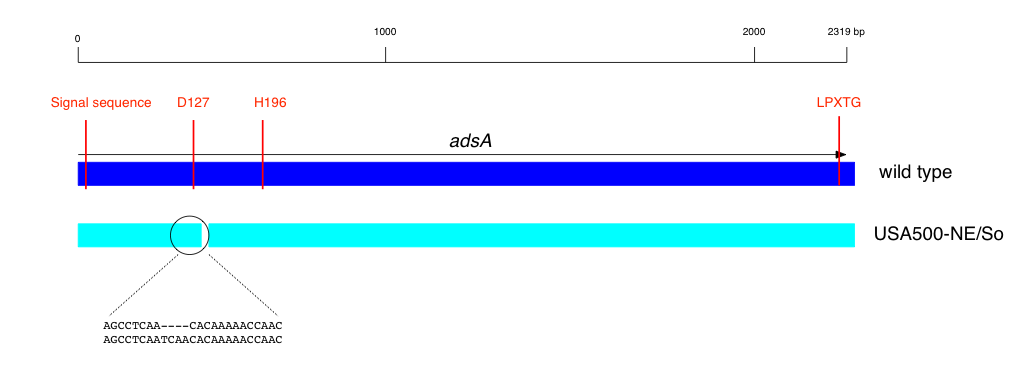


##### 
