# Supplementary material for: Invasive Methicillin-Resistant Staphylococcus aureus USA500 Strains from the U.S. Emerging Infections Program Constitute Three Geographically Distinct Lineages
Source: mSphere. 2018 May 2;3(3):e00571-17. doi: 10.1128/mSphere.00571-17 (PMC5932375; doi:10.1128/mSphere.00571-17)
Supplement: FIG S5 [file sph003182533sf5.docx]

##### Supplemental Figure 5. Phylogenetic distribution of the *adsA* frameshift mutation.

Strains with frameshift are shaded light blue on the outer ring; wild type are dark blue. Clade colors are the same as [Figure 1.](https://docs.google.com/document/d/1otCdyBq52GbYYN3pEAI5w4EujYlVmEObQ6QdpxvGzCo/edit#bookmark=id.ynmsuc7wmvn0) Only strains sequenced in this study are marked.

#####

##### 
