# Supplementary figures and images for: Invasive Methicillin-Resistant Staphylococcus aureus USA500 Strains from the U.S. Emerging Infections Program Constitute Three Geographically Distinct Lineages
Source: mSphere. 2018 May 2;3(3):e00571-17. doi: 10.1128/mSphere.00571-17 (PMC5932375; doi:10.1128/mSphere.00571-17)

##### Supplemental Figure 6. Number of strains sequenced each year (2005-2013).


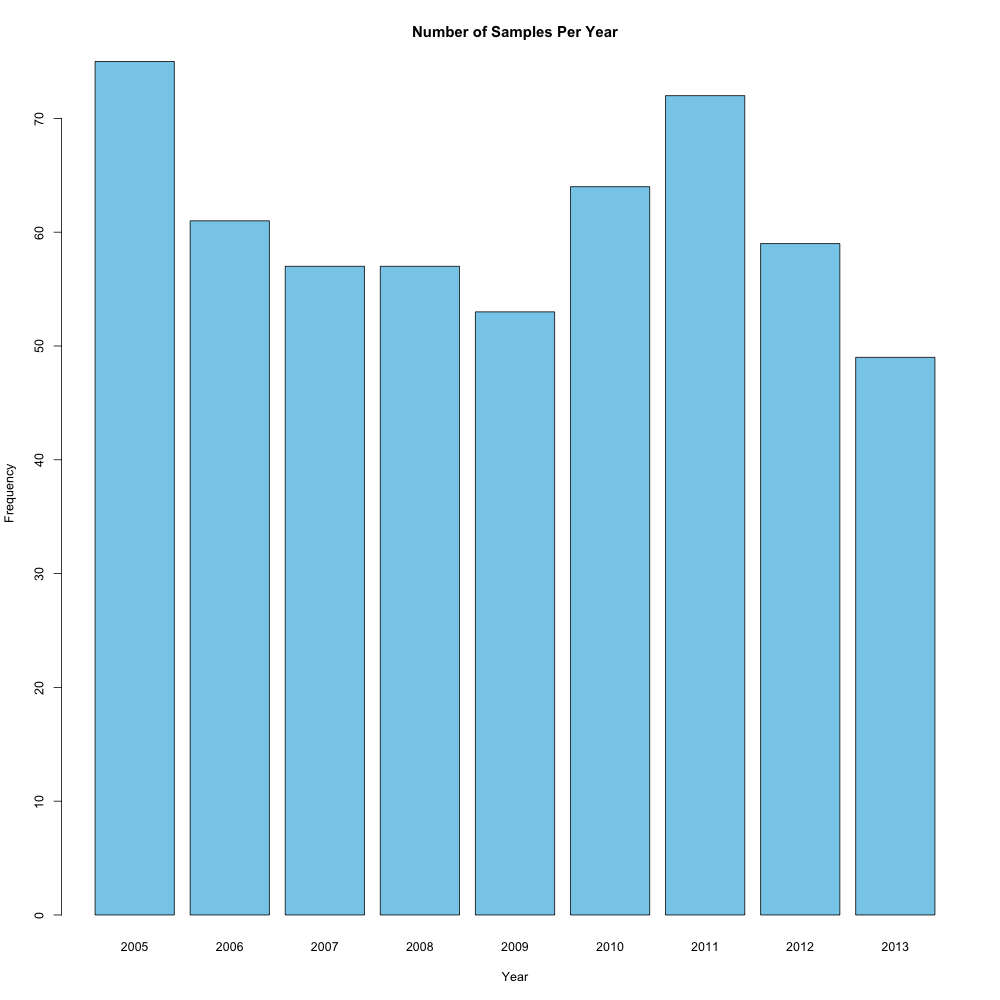


##### 

Supplement: FIG S6 [file sph003182533sf6.docx]
