# Supplementary material for: Invasive Methicillin-Resistant Staphylococcus aureus USA500 Strains from the U.S. Emerging Infections Program Constitute Three Geographically Distinct Lineages
Source: mSphere. 2018 May 2;3(3):e00571-17. doi: 10.1128/mSphere.00571-17 (PMC5932375; doi:10.1128/mSphere.00571-17)

##### Supplemental Figure 7. Convergence of molecular clock analyses

Marginal density of the tree likelihood for the two runs of the molecular clock analysis. The convergence of the two runs is clear, from the overlap of the distributions.


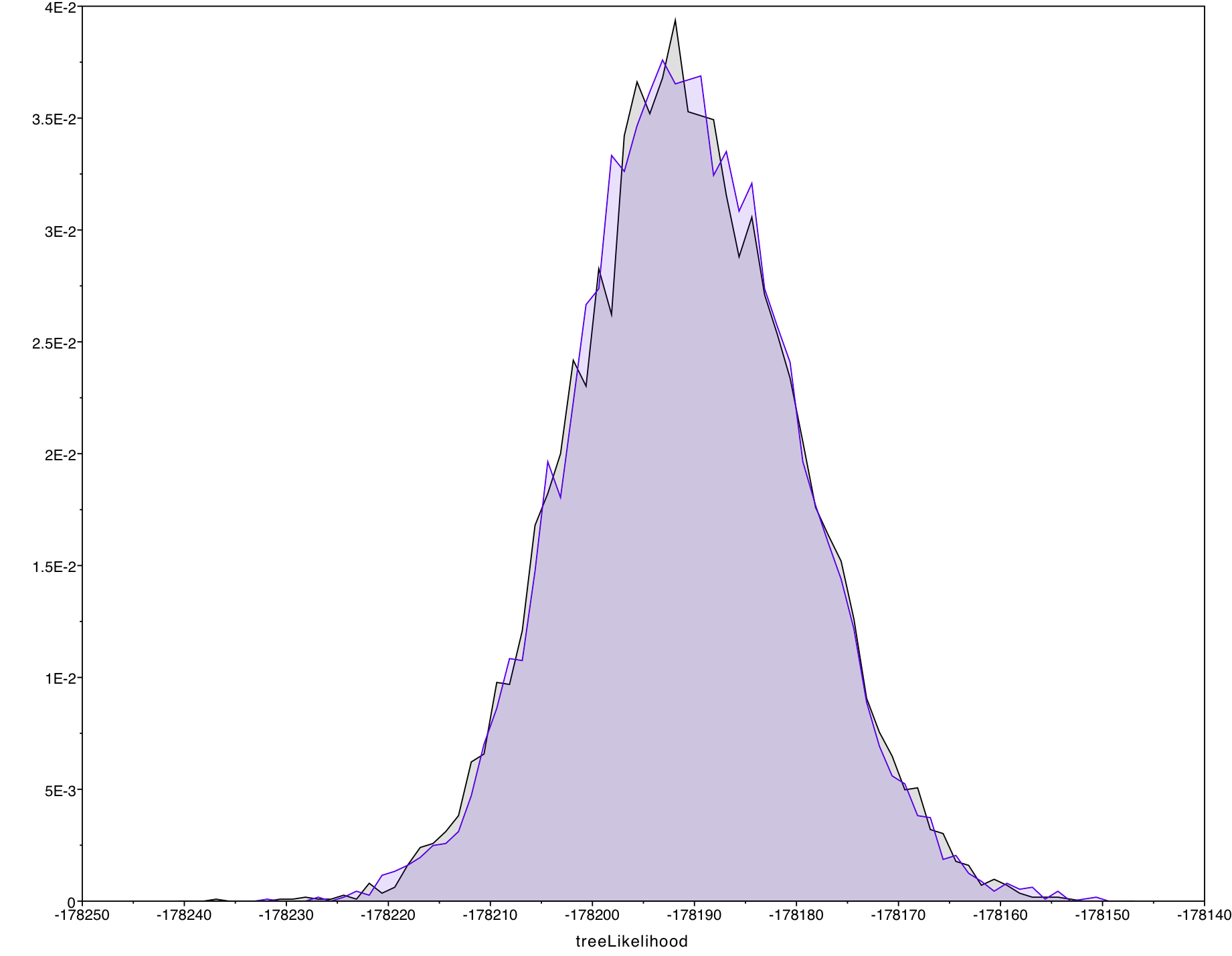

Supplement: FIG S7 [file sph003182533sf7.docx]
