# Supplementary material for: Invasive Methicillin-Resistant Staphylococcus aureus USA500 Strains from the U.S. Emerging Infections Program Constitute Three Geographically Distinct Lineages
Source: mSphere. 2018 May 2;3(3):e00571-17. doi: 10.1128/mSphere.00571-17 (PMC5932375; doi:10.1128/mSphere.00571-17)
Supplement: FIG S8 [file sph003182533sf8.docx]

##### Supplemental Figure 8. Molecular clock replicate runs.

Marginal posterior density of the tree height for 3 replicates runs (blue, orange and red) and the final molecular clock analysis (black). The marginal densities of the three replicates extensively overlap with that of the final analysis


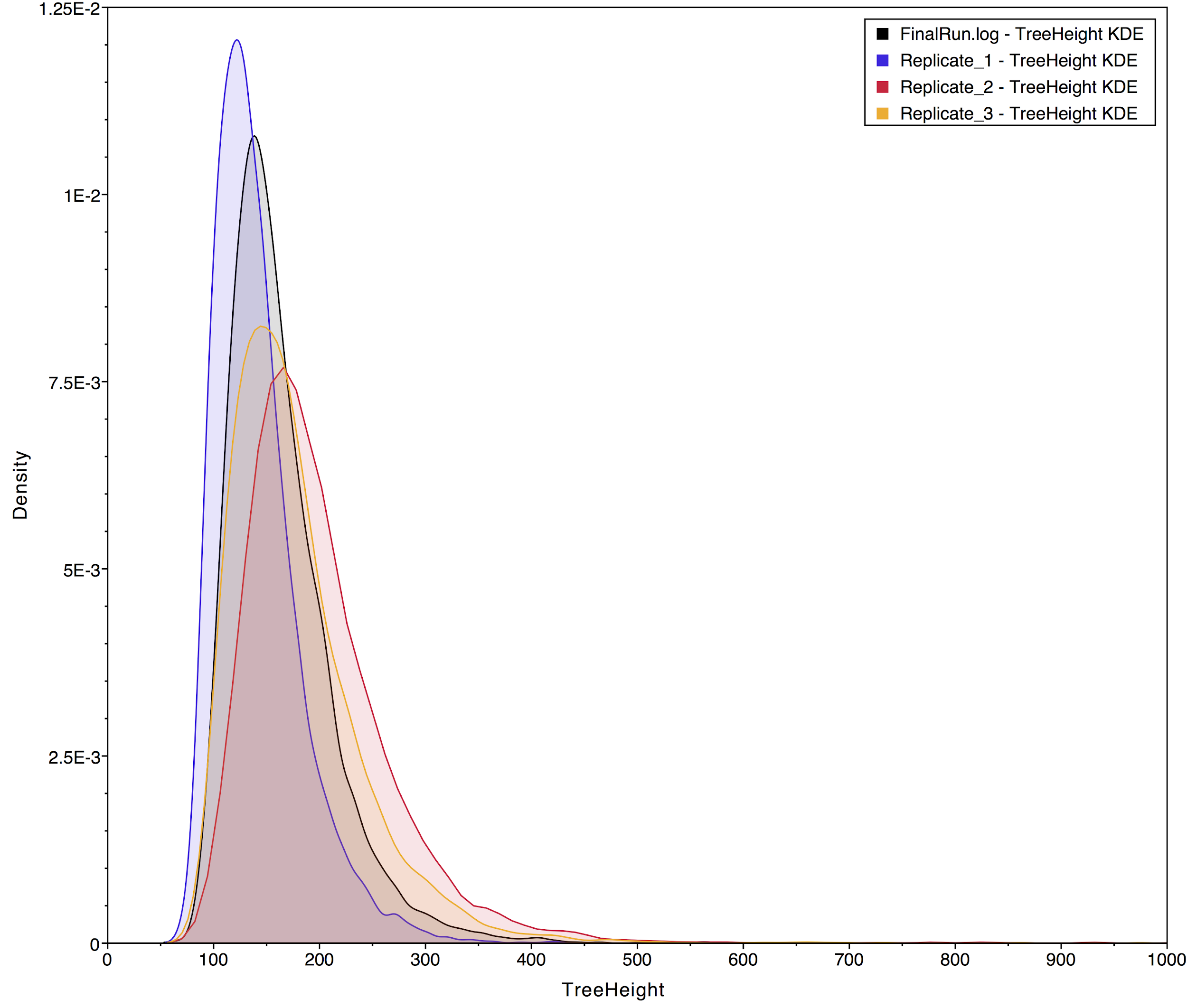


##### 
