# Supplementary material for: Invasive Methicillin-Resistant Staphylococcus aureus USA500 Strains from the U.S. Emerging Infections Program Constitute Three Geographically Distinct Lineages
Source: mSphere. 2018 May 2;3(3):e00571-17. doi: 10.1128/mSphere.00571-17 (PMC5932375; doi:10.1128/mSphere.00571-17)
Supplement: TABLE S2 [file sph003182533st2.docx]

##### Supplementary Table 2

Convergence of the two molecular clock analysis runs. The table shows similar estimates estimates of the tMRCA for the three major clades in this study for each run.

| Clade | First run | Second run |
| --- | --- | --- |
| C1 | 1936.6474 (1873.4643-1973.3851) | 1936.8894 (1877.3503 -1975.9442) |
| C2 | 1944.8372 (1890.4467-1978.5006) | 1944.3494 (1891.8237-1978.1858) |
| E1 | 1950.3324 (1898.8977-1980.0225) | 1950.5062 (1902.2744-1980.5834) |

Median estimates and 95% HPD interval in parentheses
